# The Tumor Microenvironment in SCC: Mechanisms and Therapeutic Opportunities

**DOI:** 10.3389/fcell.2021.636544

**Published:** 2021-02-09

**Authors:** Nádia Ghinelli Amôr, Paulo Sérgio da Silva Santos, Ana Paula Campanelli

**Affiliations:** ^1^Department of Biological Sciences, Bauru School of Dentistry, University of São Paulo, Bauru, Brazil; ^2^Department of Surgery, Stomatology, Pathology, and Radiology, Bauru School of Dentistry, University of São Paulo, Bauru, Brazil

**Keywords:** cutaneous squamous cell carcinoma, immunotherapy, tumor microenvironment, checkpoint inhibitors, regulatory T cell, macrophage, IL-33

## Abstract

Squamous cell carcinoma (SCC) is the second most common skin cancer worldwide and, despite the relatively easy visualization of the tumor in the clinic, a sizeable number of SCC patients are diagnosed at advanced stages with local invasion and distant metastatic lesions. In the last decade, immunotherapy has emerged as the fourth pillar in cancer therapy *via* the targeting of immune checkpoint molecules such as programmed cell-death protein-1 (PD-1), programmed cell death ligand-1 (PD-L1), and cytotoxic T-lymphocyte-associated protein 4 (CTLA-4). FDA-approved monoclonal antibodies directed against these immune targets have provide survival benefit in a growing list of cancer types. Currently, there are two immunotherapy drugs available for cutaneous SCC: cemiplimab and pembrolizumab; both monoclonal antibodies (mAb) that block PD-1 thereby promoting T-cell activation and/or function. However, the success rate of these checkpoint inhibitors currently remains around 50%, which means that half of the patients with advanced SCC experience no benefit from this treatment. This review will highlight the mechanisms by which the immune checkpoint molecules regulate the tumor microenvironment (TME), as well as the ongoing clinical trials that are employing single or combinatory therapeutic approaches for SCC immunotherapy. We also discuss the regulation of additional pathways that might promote superior therapeutic efficacy, and consequently provide increased survival for those patients that do not benefit from the current checkpoint inhibitor therapies.

## Introduction

Squamous cell carcinoma (SCC) is the second most common skin cancer worldwide and, despite the relatively easy visualization of the tumor in the clinic, a sizeable number of SCC patients are diagnosed at advanced stages with local invasion and distant metastatic lesions (Tromp et al., 2005). Worldwide, 300,000 new cases are seen each year (Thomson, 2018; Stang et al., 2019). The most significant risk factor for SCC includes sun exposure and age and is most common in white male individuals (Que et al., 2018a). Microscopically, SCC can be subcategorized according to the differentiation status of the epithelium and the presence of metastatic lesions (Que et al., 2018a). Tumor diameter and perineural involvement are highly associated with mortality risk (Que et al., 2018a). The majority of cutaneous SCC can be surgically removed, however, high risk and advanced SCC management remained to be standardized (Que et al., 2018b).

The high risk of SCC development observed in immunocompromised individuals highlights the critical role of the immune system during skin carcinogenesis (Euvrard et al., 2003; Asgari et al., 2017; Omland et al., 2018). Conversely, increased infiltration of specific inflammatory cells, such as neutrophils, macrophages, and T lymphocytes are associated with aggressive SCC and metastasis (Duan et al., 2000; Seddon et al., 2016; Jiang et al., 2019). Such discordant observations are explained by the great capacity of tumor cells to modulate the tumor microenvironment (TME) to become a supportive niche thereby inhibiting anti-tumoral responses (Weber et al., 2005; Moussai et al., 2011; Maalouf et al., 2012). The CD28-related inhibitory receptors crucial for T cell regulation, namely cytotoxic T-lymphocyte-associated protein 4 (CTLA-4) and programmed cell-death protein-1 (PD-1), are highly expressed in human SCC samples and associated with cancer progression (Welsh et al., 2009; Gambichler et al., 2017). Blocking specific immunosuppressive pathways seems to be the most promising approach to fight cancer cells. Indeed, PD-1 blockade using monoclonal antibody has been shown to increase the infiltration of CD4^+^ and CD8^+^ T cells and delays SCC development in mice (Belai et al., 2014), and the immunotherapy that blocks the signaling pathway mediated by PD-1 has been approved for cutaneous SCC. However, a significant proportion of SCC patients receive no benefit from this therapy (Migden et al., 2018). Such observations highlight the need for developing alternative or combinatory targets, that take into account the complexity of the immune system and the heterogeneity of tumor-infiltrating leukocytes in SCC (Ji et al., 2020). For example, regulatory T cells (Tregs) are observed in advanced cancers and also associated with poor outcomes (Azzimonti et al., 2015). Pre-clinical model of SCC development is also accompanied by infiltration of Tregs in the skin and draining lymph nodes (LN), and depletion of these cells using anti-CD25 significantly impaired SCC progression by increasing the infiltration of activated CD4^+^ and CD8^+^ T cells in the TME and the production of anti-tumor cytokines such as IL-12 and IFN-γ (Ramos et al., 2012). Herein, we provide a comprehensive overview of the interactions between tumor and immune cells and highlight strategies to identify potential new targets and biomarkers for immunotherapy in SCC.

## Cellular Composition of the Tumor Microenvironment

**FIGURE 1:**
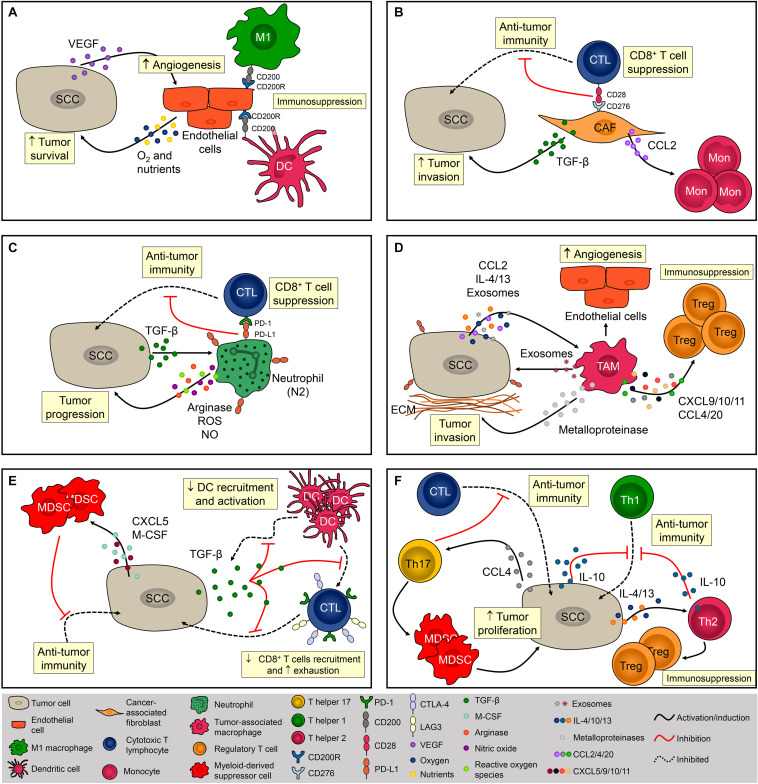
Cellular composition of the SCC microenvironment. The SCC microenvironment is comprised of cancerous and normal epithelial cells, fibroblasts, endothelial cells, and immune cells. Each one of them can exert pro- and/or anti-tumoral effects. **(A)** Tumor cells produce VEGF that promotes angiogenesis. Endothelial cells (EC) provide oxygen and nutrients that are essential for tumor cells metabolism and promote immunosuppression *via* CD200R expression. **(B)** Cancer-associated fibroblasts (CAF) impact immunosurveillance and tumor escape *via* CD276 (B7-H3), contributing to the activation of Tregs and inhibition cytotoxic CD8^+^ T cells. CAFS also promote tumor development by increasing the influx of monocytes *via* MCP-1 release and TGF-β production. **(C)** Neutrophils are recruited *via* CXCL8 chemotaxis. Neutrophils are polarized to N2 phenotype *via* TGF-β and promote SCC progression mostly by suppressing the activity of cytotoxic T lymphocytes (CTL) *via* PD-1/PD-L1 signaling. **(D)** Tumor-associated macrophages (TAM) are recruited *via* CCL2 chemotaxis and contribute to the progression of tumor by producing metalloproteinases (MMP) and recruiting regulatory T cells (Tregs). TAMs are polarized to a pro-tumor phenotype by IL-4, IL-13, and tumor-derived exosomes. **(E)** By secreting TGF-β1, SCC inhibits dendritic cells (DC) migration and the ability of DC to mature into a potent T cell activator. Tumor cells also promote immunosuppression by recruiting myeloid-derived suppressor cells *via* CXCL5 and M-CSF. **(F)** CD4^+^ T cells from a chemically-induced mouse model of SCC preferentially produce IL-4 and IL-10, promoting immunosuppression by inhibiting Th1 responses and recruiting Tregs. Th17 cells are recruited to the TME *via* CCL4 chemotaxis and promote the infiltration of myeloid cells and decrease the infiltration of IFN-γ-producing CD8^+^ T lymphocytes contributing to the immunosuppressive niche during SCC.

The SCC microenvironment is comprised of cancerous and normal epithelial cells, fibroblasts, endothelial cells (ECs), melanocytes, plasmacytoid, and dendritic cells (DCs), Langerhans cells, macrophages, myeloid-derived suppressor cells (MDSCs), natural killer (NK) cells, CD4^+^ and CD8^+^ T cells, and Tregs (Ji et al., 2020). Importantly, the frequency of each cell type varies considerably between patients, suggesting that the TME is not static and might be influenced by genetic and other patient-derived intrinsic factors (Ji et al., 2020). The role of the TME during SCC tumorigenesis and the mechanisms of immune escape are summarized in Figure 1.

The stroma adjacent to SCC is composed mainly of ECs and fibroblasts that create a fibrovascular niche (Ji et al., 2020). The association between ECs and cancer is frequently studied since angiogenesis is fundamental for SCC development (Tonini et al., 2003; Florence et al., 2011; Figure 1A). In addition, it has been demonstrated that tumor cell increased the expression of CD200 in ECs, which in combination with its ligand, CD200R (present in macrophages and DCs), might be a mechanism leading to immunosuppression in the TME (Belkin et al., 2013; Figure 1A). Fibroblasts are highly heterogeneous and multifunctional mesenchymal-derived cells embedded within the interstitial extracellular matrix that becomes activated during wound healing, tissue inflammation, and organ fibrosis (Chen and Song, 2019). Activated fibroblasts in the TME are named cancer-associated fibroblasts (CAFs) and are identified by the expression of α-smooth muscle actin (α–SMA), fibroblast-activation protein α (FAPα), and ferroptosis suppressor protein 1 (FSP-1) (Öhlund et al., 2014). CAFs directly impact the behavior of tumor cells by increasing the expression of laminin-332 γ2 chain in tumor cells through activation of the TGF-β signaling subsequently leading to enhanced cell invasion (Siljamäki et al., 2020; Figure 1B). CAFs also have a role in immunosurveillance and tumor escape *via* CD276 (B7-H3), which augments Tregs and inhibit cytotoxic CD8^+^ T cell responses (Ji et al., 2020) and promote tumor development by enhancing monocyte chemoattractant protein-1 (MCP-1)–dependent macrophage infiltration and chronic inflammation (Zhang et al., 2011; Figure 1B). However, Zhang et al. (2013a) demonstrated that CAFs can prevent carcinogen-derived tumor formation by protecting epithelial cells from DNA damage, suggesting an ambiguous role of CAFs in cutaneous SCC.

During inflammation, neutrophils are among the first phagocytes to infiltrate the tissue, mostly through CXC chemokine-mediated chemotaxis, and these cells predominate in the SCC invasive front (Kruger et al., 2015; Simonneau et al., 2018; Khou et al., 2020). Progressive infiltration of tumor-associated neutrophils (TANs) was observed during the evolution of benign papillomas to established SCC lesions in a chemical carcinogenesis model, and tumor escape mostly involved the impairment of anti-tumor CD8^+^ T cell responses mediated by high arginase activity, production of reactive oxygen species (ROS), nitrite (NO), and the induction of PD-1 expression on CD8^+^ T cells (Khou et al., 2020; Figure 1C). Similar to CAFs, TANs can also play an anti-tumoral effect in SCC. Challacombe et al. (2006) showed that neutrophil depletion increases SCC development, suggesting their role in mediating anti-tumor responses. In addition, neutrophils were necessary for the anti-tumoral effects of the Ingenol 3-angelate in experimental SCC (Challacombe et al., 2006). Such contradictory roles of these cells might be explained by the fact that, in the TME, tumor-derived factors can modulate their phenotype and function. TANs may acquire either an anti-tumor activity (N1 neutrophils), and/or a pro-tumoral activity (N2 neutrophils) mediated by TGF-b signaling (Fridlender et al., 2009; Figure 1C). In a mouse uterine cancer model, TANs exhibit N2 phenotype and promoted tumor growth through elastase release (Mahiddine et al., 2020). The authors also demonstrated that hypoxia is crucial for N2 phenotype maintenance and tumor oxygenation can revert TANs phenotype toward N1 (Mahiddine et al., 2020). Tumor-associated macrophages (TAMs) also represent a significant percentage of infiltrating phagocyte population in SCC (Kambayashi et al., 2013; Amôr et al., 2018; Simonneau et al., 2018; Jiang et al., 2019), and specific depletion of these cells inhibited tumor growth (Takahashi et al., 2009). The recruitment of monocytes into the SCC is mediated by CC chemokines such as CCL2 and, once in the TME, monocyte-derived macrophages are polarized toward a M1 or M2 phenotype (Pettersen et al., 2011; Caley et al., 2020). Due to the abundance of Th2-related cytokines such as IL-13, IL-4, and IL-10, the TME around SCC is often predominated by M2 macrophages (Linde et al., 2012b; Caley et al., 2020; Figure 1D). In addition to the TME cytokine profile, macrophages can also be M2-polarized through the secretion of tumor-derived exosomes. Exosomes derived from SCC promoted M2-like macrophage polarization through ERK1/2 signaling activation (Pang et al., 2020) besides promoting cell survival after ionizing radiation *in vitro* (Mutschelknaus et al., 2016; Figure 1D). Data from breast cancer also suggest that exosomes are capable of inducing IL-6 secretion, and a pro-survival phenotype in M2 macrophages, partially *via* gp130/STAT3 signaling (Ham et al., 2018). Interestingly, exosome-mediated communication in the TME works in both ways. Macrophage-derived exosomes increase cell migration and PD-L1 expression contributing to the establishment of an immunosuppressive TME (Bellmunt et al., 2019; Figure 1D). The contribution of TAMs directly to tumor growth occurs in early stages of carcinogenesis *via* malignant transformation and proliferation of epidermal cells. These cells also contribute to the progression of tumor by producing metalloproteinases (MMP) and increasing angiogenesis, thus facilitating the dissemination of tumor cells (Kerkelä et al., 2002; Linde et al.,2012a,b; Kambayashi et al., 2013; Figure 1D). TAMs can facilitate tumor escape by recruiting Tregs through the secretion of CXCL9/10/11 (ligands for CXCR3), CCL4 (ligands for CCR4/8), and CCL20 (ligands for CXCR3 and CCR6), which will further suppress local anti-tumoral immune responses (Ji et al., 2020; Figure 1D). Depletion of CCR2-expressing monocytes or macrophages with anti-CSF1R prevents the spontaneous development of SCC in transgenic mice (Antsiferova et al., 2017). Similarly, VEGFR-3 ligand blockade reduced SCC development by decreasing macrophage infiltration (Alitalo et al., 2013). Together, these findings strongly indicate the significant role of TAMs during SCC pathogenesis.

Given the importance of DCs in the skin, these cells are often thought to be the first immune cells to encounter tumor antigens from SCC (Valladeau and Saeland, 2005). SCC-derived DCs strongly induce CD4^+^ and CD8^+^ T-cell proliferation and IFN-γ production, thereby promoting the anti-tumoral response (Fujita et al., 2012). A significant decrease in DC infiltration has been reported in SCC and this is likely an important mechanism for tumor escape. By secreting TGF-β1, SCC inhibits DC migration and the ability of DC to mature into a potent T cell activator (Figure 1E; Halliday and Le, 2001; Weber et al., 2005). Further, *in vitro* stimulation of SCC-derived DCs showed an impaired ability of these cells to express costimulatory molecules when compared with DCs derived from normal skin, suggesting that SCC microenvironment display mechanisms that contribute to negative regulation in anti-tumor immune responses (Bluth et al., 2009). In SCC patients, this might be explained by the high expression of PD-L1 and PD-L2 in DCs (Jiao et al., 2017) or by the proximity of DCs to Tregs (Jang, 2008). Another myeloid cell that exerts a significant role in TME is the MDSCs, which promotes tumor progression by suppressing the effector function of anti-tumor immune cells from early to advanced stages of SCC (Figure 1E). Elevated circulating numbers of MDSCs were associated with high-grade SCC (Seddon et al., 2016). During early stages of tumor initiation in mice, Maalouf et al. (2012) reported an increased influx of MDSCs driven by the secretion of epidermal-derived CXCL5 and macrophage colony-stimulating factor (M-CSF) (Figure 1E). MDSCs were also found in advanced stages of SCC; these cells express high levels of CD200R^+^ that may interact with CD200^+^ tumor cells conferring immune privilege and favoring metastasis development (Stumpfova et al., 2010).

CD8^+^ T lymphocytes can directly eliminate tumor cells through the secretion of cytolytic enzymes, which are essential mediators of the anti-tumoral response (Nasti et al., 2015). However, SCC lesions display low frequencies of CD8^+^ T cells (Freeman et al., 2014) due, in part, to the presence of TGF-β, which inhibits CD8^+^ T cells infiltration and induces the expression of T cell exhaustion markers such as Tim-3, CTLA-4, and PD-1 (Weber et al., 2005; Linedale et al., 2017; Figure 1E). Blockade of PD-1 with monoclonal antibody was shown to increase tumor-infiltrating cytotoxic T lymphocytes (CTLs) resulting in anti-tumor activity against SCC growth (Belai et al., 2014), however, the depletion of CD8^+^ abrogated the efficacy of the anti-PD1 mAb treatment (Dodagatta-Marri et al., 2019), suggesting an association between the efficacy of PD-1 blockade therapies and the frequency of PD-1^+^ CD8^+^ T cells in the TME (Gros et al., 2014; Kansy et al., 2017; Kumagai et al., 2020).

Mice lacking CD4^+^ T cells and submitted to UVB-induced carcinogenesis displayed higher tumor growth associated with increased inflammation and increased number of p53^+^ tumor cells, demonstrating that this subset of T cells has an important role in controlling inflammation-associated carcinogenesis (Hatton et al., 2007). During skin carcinogenesis, CD4^+^ T cells are recruited *via* CXCL9- and CXCL10-mediated chemotaxis (Winkler et al., 2011). However, tumor cells can escape from T helper-mediated immune responses by polarizing CD4^+^ cells toward to a pro-tumor phenotype, characterized by Th2, Th17, and Treg cytokine profiles (Girardi et al., 2004; Zhang et al., 2013b; Figure 1F). IL-10-depleted mice are protected from UV-induced skin cancer due to their increased amounts of IFN-γ and enhanced numbers of CD4^+^ T cells, indicating a strong Th1-driven immune response (Loser et al., 2007). CD4^+^ T cells from a chemically-induced mouse model of SCC preferentially produced IL-4 and IL-10 (typical of Th2-type immunity) and these cells promote immunosuppression by inhibiting Th1 responses and recruiting Tregs (Yusuf et al., 2008; Nasti et al., 2011; Figure 1F). Th17 cells are recruited to the TME *via* CCL4 chemotaxis (Ortiz et al., 2015) and Wang et al. (2010) demonstrated that IL-17 contributes to skin tumorigenesis directly by increasing hyperproliferation of epithelial cells. Finally, IL-17 promoted the infiltration of myeloid cells, and decreased the infiltration of IFN-γ-producing CD8^+^ T lymphocytes contributing to the immunosuppressive niche during SCC (Wang et al., 2010; Figure 1F).

## Evasion of the Immune Response: Potential Targets for Immunotherapies

As discussed above, an effective immune response is critical to control SCC development and progression (Burton et al., 2016). To efficiently eliminate SCC, leukocytes must infiltrate the TME, initiate cell-to-cell interactions so as to exert effective anti-tumoral functions (Halliday and Le, 2001; Weber et al., 2005; Ortner et al., 2017). T cell activation involves the engagement of receptors and co-receptors (Dustin and Shaw, 1999), but this activation is countered by a series of molecules called immune checkpoints that are responsible for controlling T cell function and induce self-tolerance (Yao et al., 2013; Waldman et al., 2020).

**FIGURE 2:**
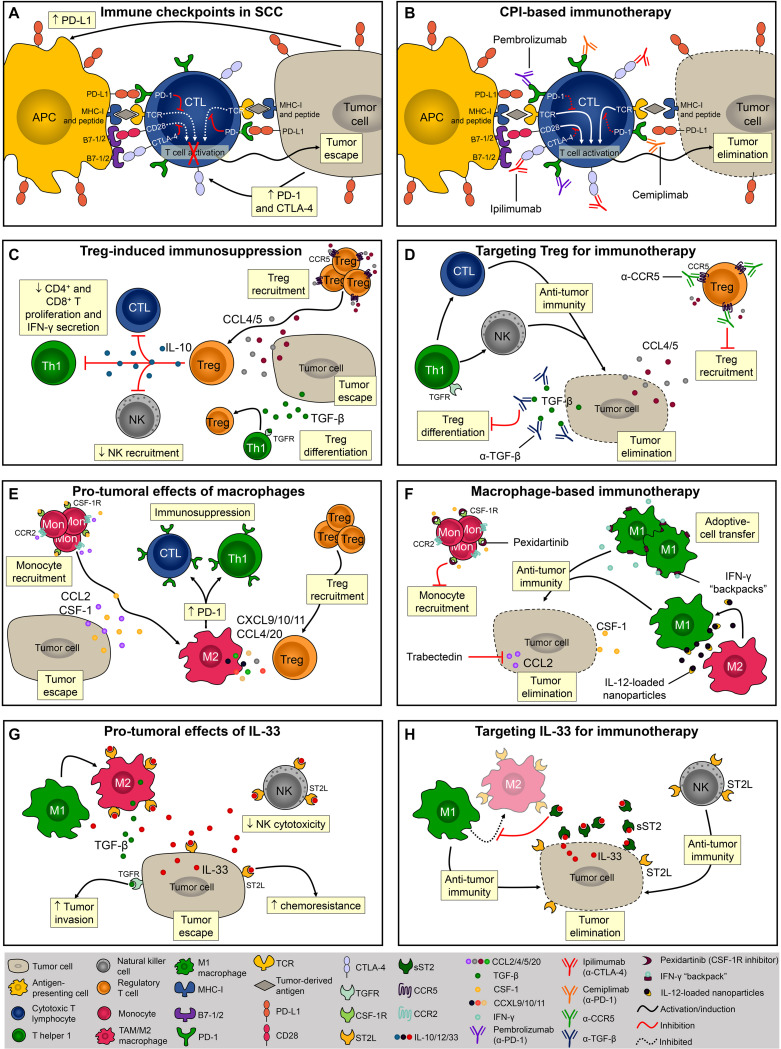
Evasion of the immune response and potential targets for immunotherapies. **(A)** *Via* the interaction between PD-1/PD-L1 and CTLA-4/B7, SCC cells inhibit CD8^+^ T cell activation and escape from immunosurveillance. **(B)** Monoclonal antibodies block PD-1 (pembrolizumab and cemiplimab) and CTLA-4 (ipilimumab) thereby promoting T-cell activation and/or function. **(C)** Tregs are recruited to TME *via* CCL4 and CCL5 or locally differentiated by TGF-β. The presence of Tregs within the TME and their levels of PD-1 expression directly correlate with immune evasion. **(D)** To overcome Treg-induced immunosuppression it is necessary to block its recruitment and/or local differentiation by inhibiting CCL4-5/CCR5 signaling or by neutralizing TGF-β. **(E)** Monocytes are recruited into the TME *via* CCL2 and CSF-1 chemotaxis. Macrophages promote SCC progression by creating an immunosuppressor microenvironment. **(F)** Pexidartinib and trabectedin are two molecules known for inhibiting macrophage recruitment into the TME. Another macrophage-based therapy involves the induction and sustained polarization of macrophages toward anti-tumoral phenotype M1. **(G)** In mice, IL-33 promotes SCC development by increasing M2 macrophage infiltration and inhibiting NK cell cytotoxicity. **(H)** Using the soluble form of the IL-33 receptor, sST2, it is possible to disrupt the IL-33/ST2 signaling and promote anti-tumor immune response mediated by M1 macrophages and NK cells.

CTLA-4 and PD-1 are the most well-studied examples of T cell immune checkpoint molecules and tumor cells exploit these molecules to evade host immunity (Miao et al., 2019). Ipilimumab, an anti-CTLA-4 blocking monoclonal antibody, was the first immune checkpoint inhibitor to be tested and approved for the treatment of cancer (Phan et al., 2003). CTLA-4 is a transmembrane molecule that competes with the co-receptors B7-1 and B7-2 for CD28 binding and negatively regulates T cell activation (Brunet et al., 1987; Linsley et al., 1992; Figure 2A). During photocarcinogenesis, mice treated with anti-CTLA-4 developed significantly fewer tumors (Loser et al., 2005). Moreover, mice treated with anti-CTLA-4 developed long-lasting protective immunity, and *in vitro* CTLA-4 blockade inhibited the suppressor activity of UV-induced Tregs, suggesting that anti-CTLA-4-treated mice were protected from tumor growth due to the inhibition of Treg function in the TME (Loser et al., 2005; Figure 2A). Despite the beneficial effects of this therapy been observed in pre-clinical models of carcinogenesis, and in patients with melanoma (Hodi et al., 2010), there is presently only one ongoing (recruiting) clinical trial assessing the efficacy of ipilimumab in cutaneous SCC (NCT04620200).

T cell activation is also regulated by PD-1 and is mediated by the interaction with its ligands, PD-L1 and PD-L2 (Freeman et al., 2000; Latchman et al., 2001), both of which are highly expressed in SCC (Belai et al., 2014; Varki et al., 2018). Blocking PD-1 resulted in a potent anti-tumoral response in a chemically induced SCC model characterized by the infiltration of activated CD4^+^ and CD8^+^ T cells, IFN-γ levels, and reduced levels of the immunosuppressor cytokine TGF-β (Belai et al., 2014; Figure 2B). Currently, two FDA-approved immunotherapies for SCC (i.e., pembrolizumab and cemiplimab) target the interaction between PD-1/PD-L1 molecules. Despite reports of adverse events in this trial, approximately half the patients with advanced SCC responded to cemiplimab therapy (Migden et al., 2018). Immune checkpoints-based therapies are of great relevance especially for patients with advanced and metastatic disease that are not ideal candidates for surgical excision. A retrospective analysis showed that PD-1 inhibition produces durable responses among patients with advanced or metastatic SCC (In et al., 2020). In a multicenter study, the overall response rate obtained among patients with advanced SCC was even higher (Salzmann et al., 2020). In both studies, the dose used was well tolerated and the response rate satisfactory, therefore adding evidence and encouraging the implementation of immune checkpoint therapies for patients with advanced and unresectable SCC lesions.

Besides T cell exhaustion, SCC development is also associated with the generation of effector Tregs (Clark et al., 2008). Tregs are characterized by CD4, CD25, and transcription factor forkhead box P3 (FOXP3) expression and, once activated, these cells suppress exacerbated immune responses and maintain self-tolerance (Sakaguchi et al., 2010; Figure 2C). The depletion of CD25^+^ Tregs significantly reduced SCC development in mice (Ramos et al., 2012). Further, the presence of Tregs within the TME and the levels of PD-1 expression by Tregs directly correlate with immune evasion (Kumagai et al., 2020) and worse outcome (Azzimonti et al., 2015). Increased percentage of tumor-infiltrating Tregs was associated with loss of inflammasome activation, and SCC development in mice (Gasparoto et al., 2014). Tregs can be either locally differentiated by TGF-β (Chen et al., 2003) or recruited *via* the CCL4/CCL5-CCR5 axis (de Oliveira et al., 2017; Figure 2C). Tregs inhibitory function is mediated by IL-10 (Loser et al., 2007), and by the inhibition of CD4^+^ and CD8^+^ T cells proliferation and IFN-γ secretion (Lai et al., 2016; Figure 2C). Importantly, in mice, anti-TGF-β monotherapy was more efficient (approximately 20% of complete regression) than anti-PD-1 monotherapy (<3% of complete regression) and promoted long-term immunity against SCC (Dodagatta-Marri et al., 2019), therefore inhibition of Tregs recruitment or differentiation using antagonistic antibodies against CCR5 or neutralizing antibodies against TGF-β are relevant strategies for immunotherapy (Figure 2D). Moreover, although anti-PD-1 monotherapy elevates immunosuppressive Tregs in chemically induced SCC, 60% of complete regression in established tumors was achieved when combined with anti-TGF-β therapy, highlighting the benefits of combinatory immunotherapies for SCC (Dodagatta-Marri et al., 2019). It has recently been demonstrated that PD-1 blockade induces both recovery of dysfunctional PD-1^+^ CD8^+^ T cells and enhanced PD-1^+^ Treg cell-mediated immunosuppression (Kumagai et al., 2020). This study suggests that the balance of PD-1 expression between CD8^+^ effector T cells and Tregs in the TME should be considered as a clinically meaningful biomarker to predict the efficacy of PD-1-blocking immunotherapy in various cancers including SCC (Aksoylar and Boussiotis, 2020; Kumagai et al., 2020).

Another recently emerging area in cancer immunotherapy has focused on macrophages since these cells frequently infiltrate solid tumors, including SCC, and have a significant impact on prognosis (Takahara et al., 2009; Li, 2016; Figure 2E). Indeed, colony-stimulating factor 1 (CSF-1) is a key regulator of monocyte/macrophage recruitment and differentiation in the TME and promotes malignancy (Lin et al., 2001; Figure 2E). In a syngeneic mouse model of melanoma, pexidartinib, a potent inhibitor of the CSF-1 receptor (CSF-1R), conferred anti-tumoral response associated with TAMs reduction (Mok et al., 2014; Figure 2F). Pexidartinib is approved for the treatment of tenosynovial giant cell tumor (Lamb, 2019) and is currently being tested in several clinical trials, none of them for SCC but with great potential (Benner et al., 2020). CCL2, also known as MCP-1, is another monocyte chemoattractant that promotes carcinogenesis by recruiting TAMs and inducing immune evasion through PD-1 signaling (Yang et al., 2020; Figure 2E). A phase II trial of trabectedin, a small molecule that specifically inhibits CCL2 synthesis, showed effectiveness as a single agent in platinum-sensitive patients with advanced recurrent ovarian cancer (Krasner et al., 2007; Figure 2F). Although trabectedin has been used to treat ovarian and breast cancer as well as soft tissue sarcomas (D’Incalci and Zambelli, 2016), its effect against SCC has yet to be investigated. While inhibition of macrophage recruitment might seem promising, M1-polarized macrophages exert potent anti-tumoral immune responses. Accordingly, M1-polarization of TAMs reversed the immunosuppressive state of TAMs and promoted tumor regression in models of ovarian cancer, melanoma, and glioblastoma (Zhang et al., 2019; Figure 2F). However, macrophage-based therapies remain highly challenging due to the plasticity of these cells (i.e., M1 macrophages can easily switch for M2 phenotype upon stimulus) (Linde et al., 2012a). To overcome this, sustained M1 differentiation is required and nanoparticles containing IL-12 promote macrophage conversion from the M2 to the M1 phenotype in the TME. Notably, this strategy has protected mice from melanoma development (Wang et al., 2017; Figure 2F). Shields et al. (2020) also successfully demonstrated that sustaining the M1 phenotype in the TME effectively controlled melanoma progression. Interestingly, this research team developed discoidal particles called “backpacks” that attach to macrophages and constantly deliver IFN-γ to sustain the M1 phenotype (Shields et al., 2020; Figure 2F).

Lastly, but still of considerable importance, soluble components of the TME, such as cytokines, chemokines, and growth factors exert a critical role during tumorigenesis (Dranoff, 2004). For example, IL-33 is abundantly expressed in epithelial cells and several other cell types (Hammad and Lambrecht, 2015), and as such it appears to be a great target for cutaneous SCC therapy. Due to its central role in mediating type 2 innate and adaptive immunity *via* the ST2 receptor, IL-33 has been extensively studied in cancer and inflammatory diseases (Liew et al., 2016). In mice, IL-33 promotes SCC development by increasing M2 macrophage infiltration and decreasing NK cell cytotoxicity (Amôr et al., 2018; Figure 2G). IL-33 promotes differentiation of macrophages that in turn, send paracrine TGF-β signals to tumor cells consequently inducing invasive behavior (Taniguchi et al., 2020; Figure 2G). In addition, IL-33 can acts directly on tumor cells to enhance chemoresistance (Fang et al., 2017), highlighting that IL-33/ST2 targeting should be considered further for SCC therapies (Figure 2G). One possible way to disrupt the IL-33/ST2 signaling is *via* the soluble form of the IL-33 receptor, sST2, which acts as a decoy receptor and prevents the binding of IL-33 to ST2L on the cell surface (Hayakawa et al., 2007; Griesenauer and Paczesny, 2017; Figure 2H). Although the effects of sST2 administration have not been tested in SCC, its potential therapeutic benefits were demonstrated in colorectal carcinoma (Akimoto et al., 2016). The reduced tumor growth observed in sST2-treated mice was associated with decreased angiogenesis, and inhibition of macrophage infiltration and M2 polarization (Akimoto et al., 2016; Figure 2H).

## Concluding Remarks and Perspectives

Growing evidence highlights the crucial contribution of immune and non-immune cells during SCC pathogenesis, most notably in the TME, and the targeting of this supportive tumor niche is an important part of emerging therapies in this cancer. As the understanding of the mechanistic events that permit tumor evasion from immunosurveillance emerges, comprehensive treatment methods that enhance anti-tumor immunity and the sensitivity of tumor cells to chemotherapies will revolutionize the therapy landscape in SCC. Although emerged as the advent for cancer treatment, one of the major limitations of immunotherapy is the development of acquired resistance to treatment due to triggering of compensatory mechanisms resulting in tumor relapse/progression. For example, in SCC, the anti-PD-1 treatment that is supposed to reduce T cell suppression also promotes the infiltration of Tregs, which are known to directly assist tumor escape in experimental SCC (Dodagatta-Marri et al., 2019). Similarly, in head and neck SCC (HNSCC), PD-1 blockade culminated in Tim-3 upregulation, supporting a circuit of compensatory suppressor signaling allowing tumor escape (Shayan et al., 2017). Therefore, combinatory therapies are the more promising future strategies in SCC therapy. Clinical data have shown that melanoma patients treated with anti-PD-1 and anti-CTLA-4 monoclonal antibodies (mAb) presented tumor reduction (Wolchok et al., 2013; Postow et al., 2015). Similarly, simultaneous blockade of GITR and PD-L1 demonstrated safety and efficacy against advanced solid tumors (Heinhuis et al., 2019). Results from over 50% of patients with recurrent or metastatic HNSCC have shown disease stabilization after treatment with motolimod, an agonist of TLR8 which stimulates NK, DC, and monocytes, in combination with cetuximab in a phase Ib clinical trial (Chow et al., 2017). Altogether, these studies represent an exciting new horizon that could be also tested for cutaneous SCC treatment.

Additionally, the next-generation sequencing (NGS) technology has emerged as one of the most powerful tools for cancer research since enables efficient and accurate detection of somatic mutations frequently associated with treatment resistance (Łuksza et al., 2017). Using the NGS approach, Lobl et al. (2020) identified the co-occurrence of ERBB4 and STK11 mutations in localized cutaneous SCC, which prompted the researchers to suggest a new therapeutic approach by inhibiting CDH1 and the Wnt pathway. Besides identification of tumor mutational burden, NGS also allows the prediction of response and development of a personalized treatment that might significantly improve outcomes for cancer patients (Holbrook et al., 2011; Roychowdhury et al., 2011; Chen et al., 2019). Based on that, NGS could and should be incorporated to assess mutations not only in tumor cells but also in immune cells of SCC patients that will help to overcome the differences of response rate observed among patients with similar malignancy and treatment, leading to the development of personalized therapies improving clinical outcomes.

Herein, we provide a range of alternative targets focused on the regulation of immune cells beyond T cells to promote anti-tumoral responses. However, a reasonable amount of the work regarding immunological therapeutic strategies remains to be evaluated against SCC, highlighting opportunities for therapeutic intervention. Since the composition of the TME is heterogeneous and result in different TME subclasses not only among patients or tumor types but also within a patient’s tumor, it is important to consider the differences in the spatial localization, density, and functional orientation of immune cells in the TME, in order to predict and improve the clinical benefits [reviewed extensively by Pérez-Ruiz et al. (2020)]. Most exciting to us are experimental strategies around macrophage- and/or IL-33-based therapies in SCC. We concede that most of the data regarding these strategies are derived from experimental models with little clinical insight, but animal models do provide key early insights into the role of immune cells and soluble factors in SCC tumorigenesis. We contend that these limitations will be lessened as further translational investigations using human subjects and clinical trials are implemented to better assess the efficacy of alternative strategies for SCC immunotherapy.

## Author Contributions

NA contributed to graphic drawing and drafting of the manuscript. PS contributed to drafting of the manuscript. AC contributed to concept generation and drafting of the manuscript. All authors contributed to the article and approved the submitted version.

## Conflict of Interest

The authors declare that the research was conducted in the absence of any commercial or financial relationships that could be construed as a potential conflict of interest.
